# Supplementary material for: Deep learning-based optical field screening for robust optical diffraction tomography
Source: Sci Rep. 2019 Oct 23;9:15239. doi: 10.1038/s41598-019-51363-x (PMC6811526; doi:10.1038/s41598-019-51363-x)
Supplement: Supplementary file 1 — Supplementary Information to Deep learning-based optical field screening for robust optical diffraction tomography [file 41598_2019_51363_MOESM1_ESM.pdf]

Supplementary Information

Deep learning-based optical field screening for robust optical diffraction tomography

DongHun Ryu[a,b], YoungJu Jo[a,b,c,d], Jihyeong Yoo[c], Taean Chang[a,b], Daewoong Ahn[c],Young Seo Kim[a,b,c],Geon Kim[a,b], Hyun-Seok Min[c], and YongKeun Park[a,b,c,*]

[a]Department of Physics, Korea Advanced Institute of Science and Technology (KAIST), 34141 Daejeon, Republic of Korea

[b]KAIST Institute for Health Science and Technology, 34141 Daejeon, Republic of Korea

[c]Tomocube, Inc., 34109 Daejoen, Republic of Korea

[d]Department of Chemical and Biomolecular Engineering, KAIST, 34141 Daejeon, Republic of Korea

[e]Present address: Department of Applied Physics, Stanford University, Stanford, CA 94305, USA

*yk.park@kaist.ac.kr

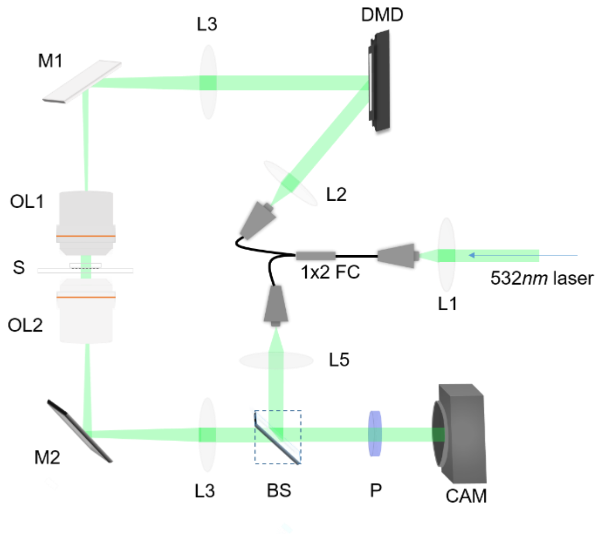

Figure S1. ODT imaging system. L: Lens, FC: Fiber coupler, DMD: Digital micromirror device, M: Mirror, BS: Beam splitter, OL: Objective lens, S: Sample, P: Linear polarizer, and CAM: Camera.

We used a commercial ODT system (HT-1S, Tomocube Inc.) with custom modifications (Fig. 6). The system is based on Mach-Zehnder interferometry. We used a diode-pumped solid-state laser beam (532 nm wavelength, 10 mW, MSL-S-532-10 mW, CNI laser, China) coupled into a 1×2 fibre coupler (OZoptics, Canada) that outputs a sample and a reference beam. The upward sample beam passing through Lens 2 is incident on a DMD (DLP6500FYE, Texas Instruments, USA) that generates many orders of diffraction beams, which was used to control the illumination angle. The first-order diffracted beam, passing through lens 3 and objective lens 1 (Numerical aperture (NA) = 0.7, ×60), impinges on a sample. Next, the scattered sample signal, conveyed by Objective lens 2 (×60, NA = 0.8) and the reference beam generate an off-axis hologram at the complementary metal-oxide-semiconductor camera (FL3-U3-13Y3M-C, FLIR Systems, USA). The captured holograms are utilised to reconstruct the 3D RI tomogram, according to the explained procedure in the main text.

The ODT imaging system, which was used to obtain NIH3T3 data, have several different optical components to the system that acquired the bacteria dataset; laser beam (632 nm wavelength, 15 mW, Thorlabs Inc., USA), an objective lens (NA = 1.1, LUMPLN, 60×, Olympus Inc., Japan), and an condenser lens (NA = 1.2 UPLSAPO, 60×, Olympus Inc., Japan).

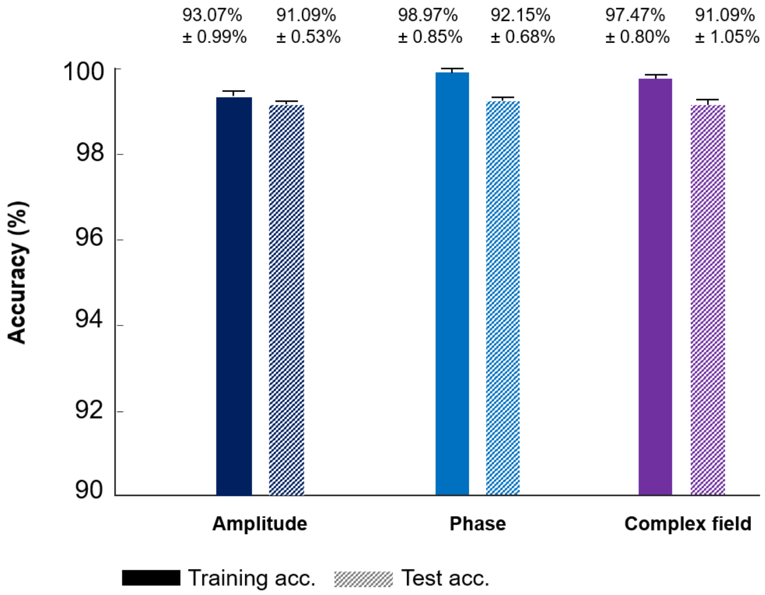

Figure S2. Training and test accuracy of classification network using amplitude, phase, and complex field data.

We decided to use phase images as input to our network, which achieved the best training and test accuracy in comparison to the results using amplitude and complex field concatenating amplitude and phase. As for the described bacteria dataset, the training accuracy for amplitude, phase and complex field are 93.07 ± 0.99%, 98.97 ± 0.85%, and 97.47 ± 0.80%; the test accuracy for the cases are 91.09 ± 0.53%, 92.15 ± 0.68%, and 91.09 ± 1.05%. Though these accuracies rely on the complexity of network architecture, we anticipate that accuracy for the classification network trained using phase dataset would be higher than amplitude or complex field for most of the low-absorption samples.

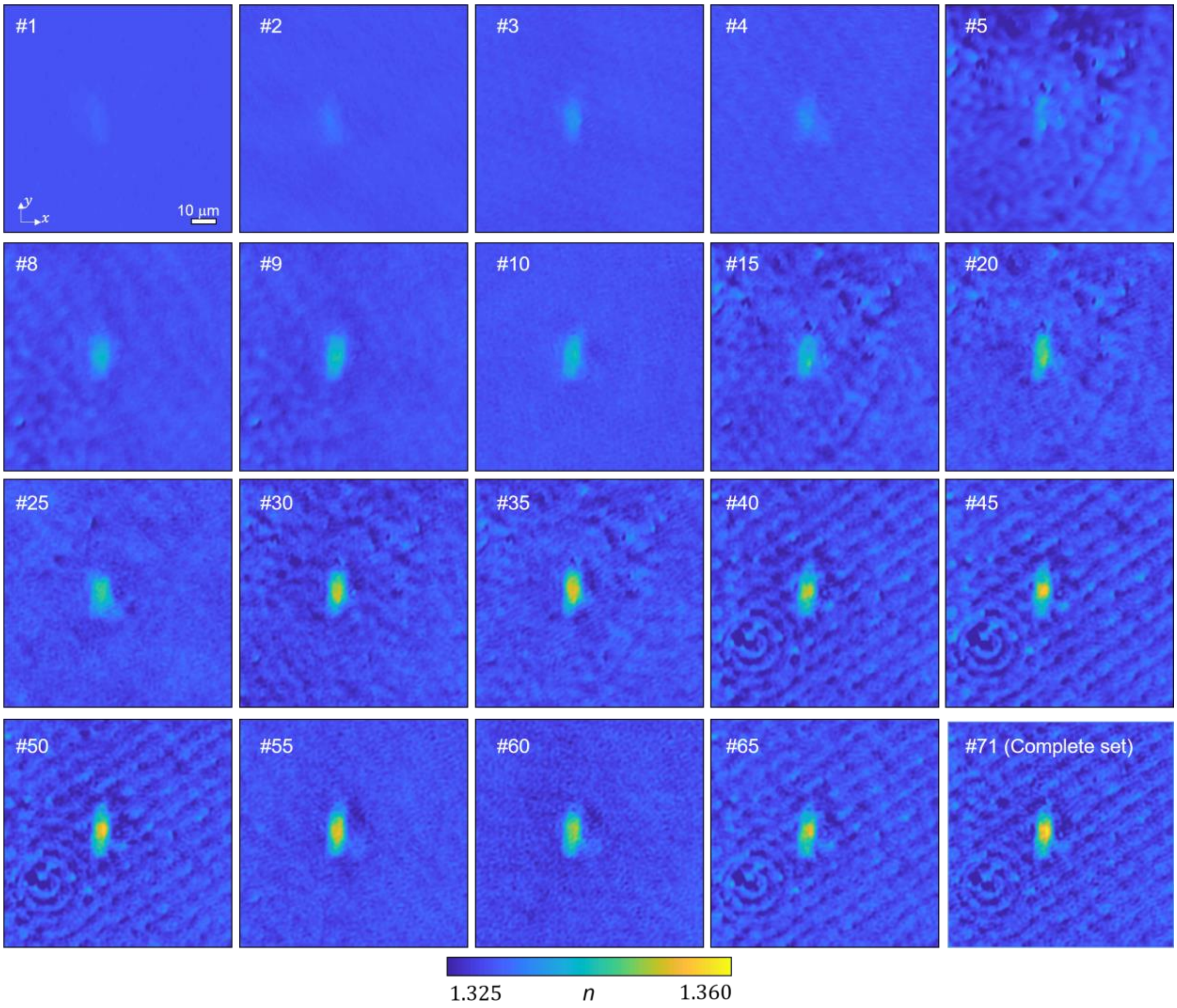

Figure S3. 2D sliced image of reconstructed tomogram of *E. coil* as a function of number of illuminations. #n indicates the number of the optical fields used for the reconstruction and each input set was generated by random sampling (from the uniform distribution) of optical fields (the number of the complete set = 71).

Acceptable quality of tomogram can be reconstructed from 30 of illumination angles, based on the resolved subcellular feature of *E. coil* and its contrast to the background. However, random combination that include noisy optical fields definitely degrade the image quality of the reconstructed tomograms. It ascertains the importance of the field screening process for robust ODT.

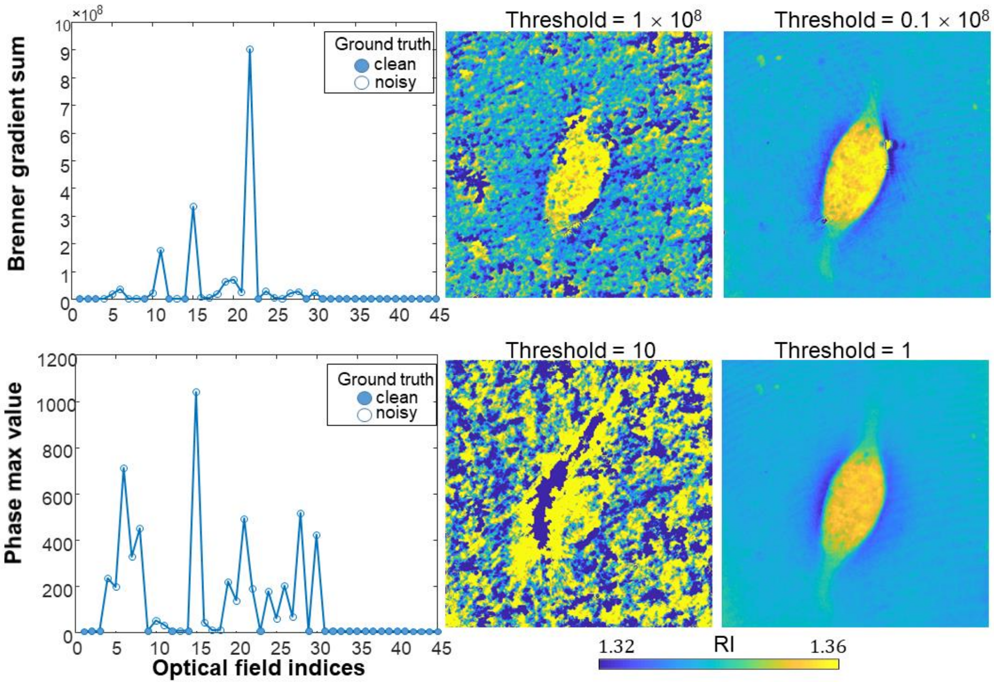

Figure S4. Additional results of rule-based algorithms using phase gradient between neighboring pixels (row 1) and phase max (row 2). Depending on thresholding values, the classification performance significantly changes, and the tomogram reconstruction quality subsequently does. We believe algorithms with carefully designed metric could result in good classification performance when fine-tuning of parameters for known data distribution incorporated.
